# Selection methods for proximity-dependent enrichment of ligands from DNA-encoded libraries using enzymatic fusion proteins†

**DOI:** 10.1039/d2sc05495g

**Published:** 2022-11-15

**Authors:** Bo Cai, Amol B. Mhetre, Casey J. Krusemark

**Affiliations:** a Department of Medicinal Chemistry and Molecular Pharmacology, Purdue Center for Cancer Research, Purdue University West Lafayette IN 47907 USA cjk@purdue.edu

## Abstract

Herein, we report a selection approach to enrich ligands from DNA-encoded libraries (DELs) based on proximity to an enzymatic tag on the target protein. This method involves uncaging or installation of a biotin purification tag on the DNA construct either through photodeprotection of a protected biotin group using a light emitting protein tag (nanoluciferase) or by acylation using an engineered biotin ligase (UltraID). This selection does not require purification of the target protein and results in improved recovery and enrichment of DNA-linked ligands. This approach should serve as a general and convenient tool for molecular discovery with DELs.

## Abstract 2

Nanoluciferase or engineered biotin ligase fusions to a protein target allow proximity-induced biotinylation of DNA-linked ligands. The approach benefits ligand enrichment from DNA-encoded chemical libraries (DELs) and live cell selections.

## Graphical abstract

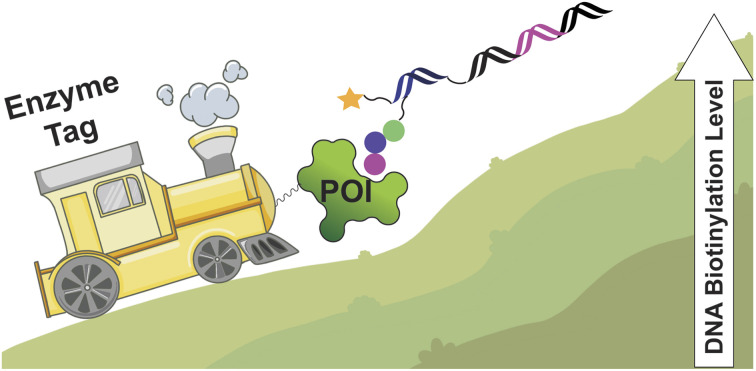

## Introduction

DNA-encoded chemical libraries (DELs) have become a fertile source of novel bioactive small molecules.^1–3^ In a typical DEL selection, a library is incubated with a protein target of interest that is immobilized on a solid support. Subsequent washing of the support, elution, and DNA sequencing allows the differentiation of binders over non-binders. While solid phase-based selections have been successfully used in a variety of DEL campaigns, some proteins are not amenable to recombinant expression and purification or may lose native structure and activity during immobilization. In addition, this traditional approach often does not generate an adequate signal-to-noise ratio to identify ligands, particularly for weak ligands from highly complex libraries.^4^

To address these limitations, several strategies have been developed, including performing iterated DEL selections against non-immobilized proteins through exonuclease digestion,^5^ the binder trap enrichment approach,^6,7^ interaction-dependent PCR,^8^ separation of target-ligand complexes in kinetic capillary electrophoresis,^9^ dynamic DNA hybridization approach,^10,11^ and affinity labeling.^12,13^ Among these methods, covalent crosslinking by affinity labelling has demonstrated remarkable properties, particularly in the enrichment of low-affinity ligands^12,14^ and in the use of non-immobilized and unpurified protein targets.^5,13^ Recently, the application of affinity labelling has extended the DEL approach to both endogenous and recombinantly expressed targets on and inside live cells.^15,16^

While generally successful, we have encountered two particular limitations to the covalent crosslinking approach. First, the requirement of purification of the DNA–protein conjugate following the crosslinking step can lead to low recovery of the DNA-linked ligand.^15^ This is particularly the case for integral membrane target proteins, which are challenging to solubilize and purify. Second, the crosslinking efficiency of target proteins to the DNA-linked ligand is generally modest (typically around 10% when binding is saturated) and can be quite low in some cases, which similarly results in low recovery. Also, this efficiency is proportional to the fraction of the DNA-linked ligand that is bound to a protein. Thus, in cases of low affinity ligands or cases of target proteins at low concentration (such as live cell conditions), crosslinking efficiency is inherently low.

We sought to explore alternative approaches that could overcome these limitations of crosslinking, while at the same time maintain the ability to assay DEL ligands binding to proteins while in solution and within complex mixtures, such as lysates or live cells. We were inspired by recent genetic approaches for determination of protein–protein and protein–nucleotide interactions *via* enzyme-mediated proximity labelling (BioID, APEX, *e.g.*).^17^ Similarly, previously demonstrated selection approaches for DNA-encoded enzyme substrates can capitalize on enzymatic turnover to effectively enrich low affinity ligands.^18^ Here, we explore two approaches for enzyme-mediated enrichment of DNA-encoded ligands. Both methods are proximity-based and effectively enrich DNA-linked ligands over non-ligands. Importantly, both methods require no protein purification, immobilization, or optimization of crosslinking efficiency.

## Results and discussion

**Fig. 1:**
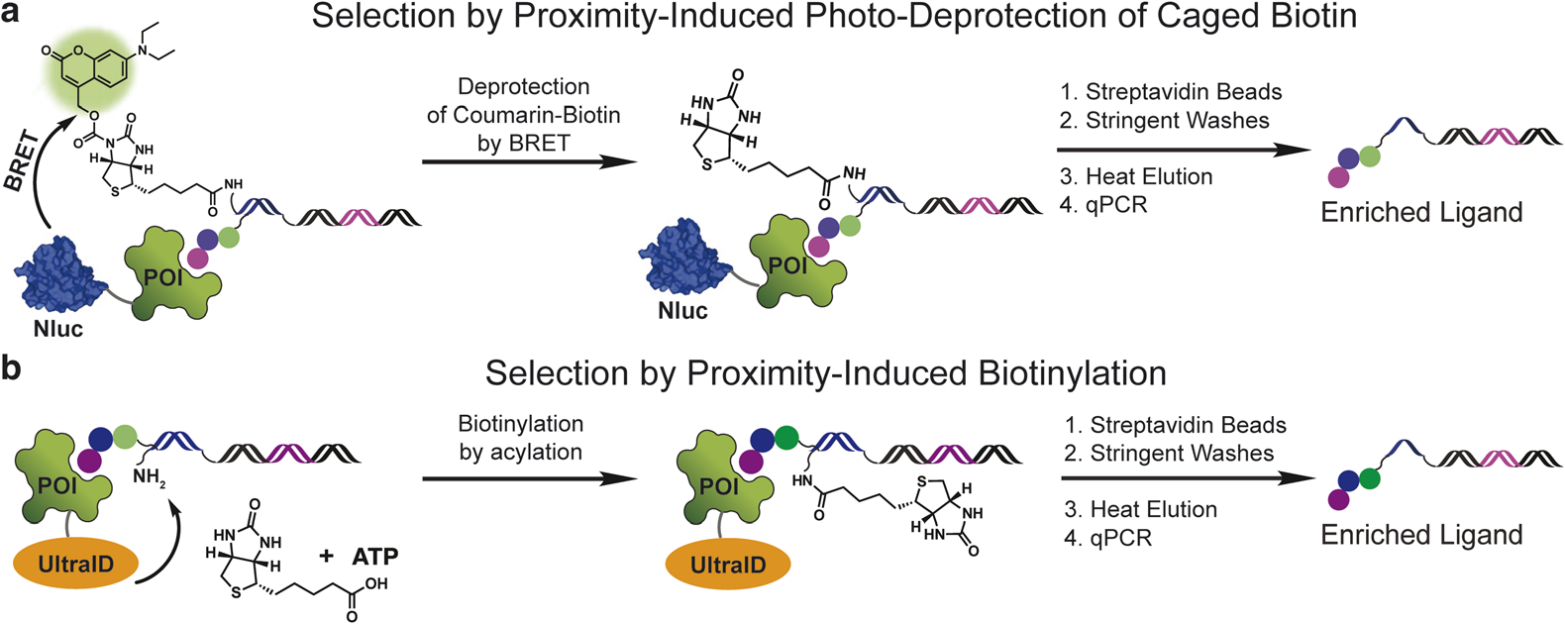
Proximity-induced selection approaches for enriching DNA-linked ligands from DNA-encoded chemical libraries. (a) Selection by proximity-induced photo-deprotection of caged biotin *via* BRET from nanoluc. (b) Selection by proximity-induced biotinylation using an engineered biotin ligase UltraID.

Initially, we explored a proximity-induced selection approach relying upon deprotection of a photocaged biotin (Fig. 1a) *via* bioluminescence resonance energy transfer (BRET) from a light emitting protein tag (Fig. 1a). We used a previously demonstrated coumarin-based photoprotecting group that can be deprotected with the light emitted from the nanoluciferase enzyme (nanoluc).^19^ As shown in Fig. 1a, a small molecule ligand is conjugated on the 5′-end of a double-stranded DNA construct, and a coumarin-caged biotin is linked to the 3′-end of the opposite DNA strand. Incubation of the ligand with a nanoluc (Nluc)-fused protein target brings the caged biotin close to Nluc, leading to proximity-induced photo-deprotection of the caged biotin. DNA-linked ligands with the exposed biotin purification tag can then be purified by streptavidin beads.

The second method capitalizes on the proximity labelling using an engineered biotin ligase. The BioID^17^ tag is a mutated version of the biotin ligase BirA^20^ and has been widely used for molecular interaction mapping with fusion proteins. BioID enables proximity labelling by releasing the biotinoyl–adenylate intermediate (a reactive acyl phosphate) from the enzyme active site, which then labels protein lysine amines non-specifically in an approximate 10 nm radius. To develop this approach for DEL selections, we used the recently developed UltraID,^21^ a BioID variant with a smaller size, faster biotinylation kinetics and, most importantly, lacks the DNA-binding domain present in BioID. In this approach, a protein target is fused with UltraID, incubated with a DNA-linked ligand with a free amine linked to the opposite DNA strand (Fig. 1b). The binding event between the protein and ligand leads to proximity-induced biotinylation of the amine modified DNA. The ligand can then be separated from non-ligands by streptavidin beads.

Both approaches remove the requirement of protein purification and immobilization. The effective amplification of the signal through enzymatic turnover may improve the ligand recovery and enrichment for low affinity ligands and ligands selected against target proteins under low concentration. Likewise, the strong interaction between biotin and streptavidin could improve ligand recovery and enrichment by allowing stringent washing conditions.

**Fig. 2:**
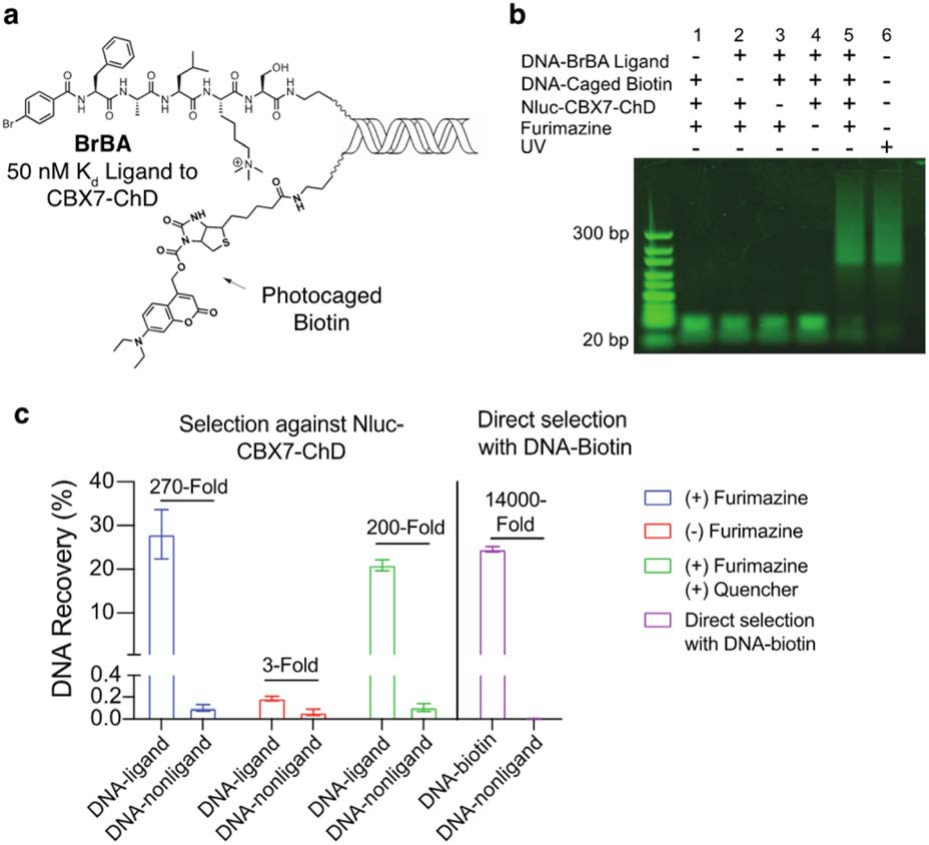
Proximity-induced photo-deprotection approach with Nluc-CBX7-ChD. (a) Structures of the DNA-linked BrBA (ligand to CBX7-ChD) and DNA-linked photocaged biotin. (b) Gel shift assay for BRET-induced uncaging of coumarin-protected biotin. DNA constructs (1 μM) were incubated with Nluc-CBX7-ChD (2 μM) in the presence of furimazine (50 μM) for 30 minutes. Prior to PAGE analysis, excess neutravidin (2 μM) was added. (c) qPCR analysis of DNA recovery and enrichment from test selections against Nluc-CBX7-ChD. Selection mixtures consisted of a ligand DNA (BrBA or biotin) (0.1 nM) and a non-ligand DNA (100 nM) mixed at a 1000 : 1 ratio. Photo selections contained 2 μM Nluc-CBX7-ChD, 50 μM furimazine substrate, and 500 μM hydrodabcyl quencher, as indicated, and were incubated for 30 minutes prior to purification on streptavidin beads and qPCR.

**Scheme 1:**
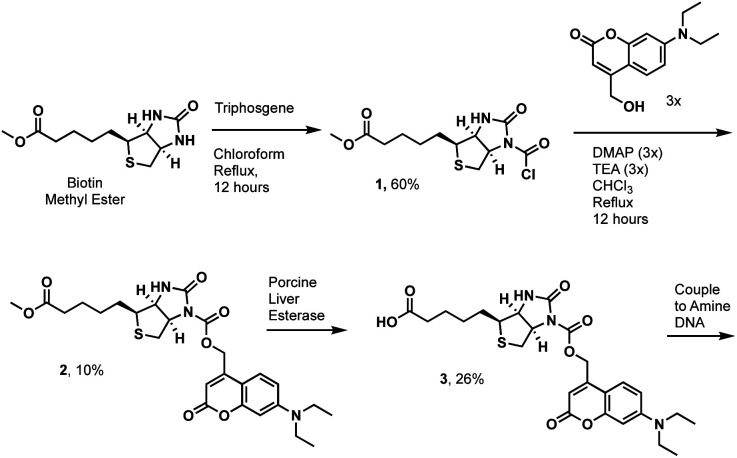
Synthesis of a photocaged biotin using a coumarin protecting group.

We first tested the proximity-induced photo-deprotection method using a model ligand-receptor system: Nluc-Chromobox Homolog 7-Chromodomain (Nluc-CBX7-ChD, Fig. S1†) and a peptidic ligand (BrBA).^22^ The BrBA ligand was conjugated to DNA *via* its C-terminus to retain binding to CBX7-ChD (Fig. 2a). We synthesized a photocaged-biotin (Scheme 1) using a coumarin protecting group linked *via* carbamylation of *N*′-1 biotin urea.^19^ Previous work has shown effective photocaging of biotin–avidin binding through analogous modification with shorter wavelength protecting groups.^23^ The coumarin–biotin (3) was conjugated to the 3′ end of a 20-mer single stranded DNA (ssDNA) by amine acylation to allow conjugation to the DNA-linked BrBA ligand *via* hybridization (Fig. 2a). The photo-deprotection of caged biotin was validated by gel shift assays *via* binding to neutravidin (Fig. 2b). Nluc-induced unmasking of the caged biotin was nearly quantitative under these conditions. The deprotection was dependent on the presence of the BrBA ligand, the caged biotin construct, the protein target, and the furimazine substrate.

We then performed a test selection assay against Nluc-CBX7-ChD using a DNA-encoded BrBA and a non-ligand control construct (Fig. 2c). Following selection, qPCR analysis was performed to quantify the recovery and enrichment of the DNA constructs. As expected, DNA-linked BrBA was highly enriched over a non-ligand control, and this enrichment was dependent on the presence of the Nluc substrate. In a control selection assay of a biotinylated DNA construct, similar recovery of biotinylated DNA was observed, suggesting high efficiency of photodeprotection (Fig. 2c); however, the fold-enrichment was ∼50-fold higher due to a reduced recovery of the nonligand-DNA. The higher background in Nluc-mediated selections could be due to background deprotection of non-ligands from ambient or Nluc-emitted light and/or hydrolysis of the caged biotin. We performed selections in the presence of a hydrodabcyl quencher that effectively absorbs light in the range of coumarin absorption, which should reduce light-based background.^24^ We initially performed gel shift assays to determine the maximum amount of quencher that would not affect desired proximity-based deprotection, and no effect was observed at 500 μM and below (Fig. S2†). In subsequent selection assays, the addition of quencher did not reduce the background signal (Fig. 2c), indicating the background deprotection of non-ligands is likely due to the hydrolysis of caged biotin over the course of the experiment. The lability of photoprotecting group may have been anticipated. In the synthesis of the protected biotin, enzymatic hydrolysis of a methyl ester was required to preserve the urea modification (Scheme 1).

To evaluate the potential of the Nluc enzymatic activity to amplify the enrichment of weak-affinity ligands, we performed a gel shift assay under sub-saturating binding conditions. We used a low affinity ligand to CBX7-ChD, 110A ref. 25 (on-DNA *K*_d_ ≈ 8 μM to CBX7-ChD, Fig. S3†) with a protein concentration (1 μM NLuc-CBX7-ChD) to yield approximately 10% of the ligand bound. To maximize the number of turnovers of Nluc with a substrate of limited solubility (∼50 μM), the deprotection was conducted in a small volume dialysis chamber within a larger volume containing buffer with 50 μM furimazine (Fig. S4†). This allows incubation with a 13 000-fold molar excess of the substrate over the Nluc-protein target. After a 6 hour incubation, the photo-deprotection of caged biotin was nearly quantitative as detected by a neutravidin gel shift assay (Fig. S4†). Significant background deprotection (≈10%) of a non-ligand control was also observed, however. Development of a photocaged biotin with greater stability is likely to reduce background deprotection rates and increase the utility of Nluc-mediated enzymatic turnover to enrich low affinity ligands. Likewise, a photoprotecting group with increased spectral overlap with Nluc emission and better photolysis efficiency would yield improved selections.^26^

**Fig. 3:**
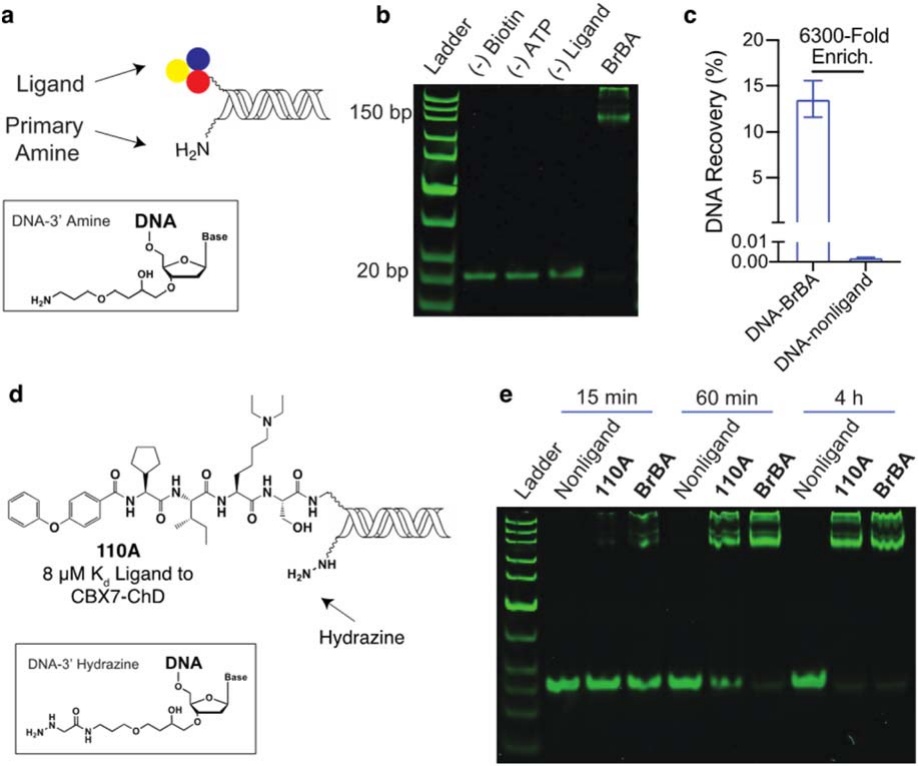
Proximity-induced biotinylation approach with UltraID-CBX7-ChD. (a) Dual presentation of a ligand and a primary amine on-DNA with inset showing 3′ linkage to an amine. (b) Gel shift assay for studying the UltraID-induced biotinylation of DNA-linked BrBA with its complementary DNA-linked amine. DNA constructs (1 μM) were incubated with UltraID-CBX7-ChD (2 μM) in the presence of biotin (50 μM), ATP (2.5 mM) and MgCl_2_ (5 mM) for 30 minutes. Prior to gel analysis, DNA was purified from the excess biotin by precipitation and incubated with neutravidin (10 μM). (c) qPCR analysis of DNA recovery from test selections against UltraID-CBX7-ChD. (d) Structures of low affinity CBX7-ChD ligand 110A paired with a hydrazine nucleophile. (e) Gel shift assay of UltraID-induced biotinylation of DNA-linked ligands (BrBA or 110A) with complementary DNA-linked hydrazine. Constructs were treated as in (b) and incubated for the indicated times.

We similarly tested the proximity-induced selection approach using the engineered biotin ligase, UltraID, as the enzymatic tag. Gel shift assays were performed using DNA-linked BrBA ligand (Fig. 3a and b) with purified UltraID-CBX7-ChD fusion protein (Fig. S1†). In this case, the BrBA ligand oligo was paired with a commercially available 3′ amine-modified oligo, as the nucleophile to react with the acyl phosphate biotin (Fig. 3a). The successful installation of biotin onto DNA was confirmed by the gel mobility shift of DNA-linked BrBA construct (Fig. 3b). A subsequent test selection against UltraID-CBX7-ChD (Fig. 1b) yielded over 6000-fold enrichment of the BrBA ligand (Fig. 3c) and was comparable to the control selection of a biotinylated construct (Fig. 2c). We additionally tested the effects of amine linker length, which would modulate the effective molarity of the amine and the UltraID tag.^27^ We found only a modest improvement with an increased linker length (Fig. S5†). We also investigated alternative nucleophiles on biotinylation efficiency (Fig. S5†) and found a marked improvement in switching from a primary amine to a hydrazine as the nucleophile.

We used the hydrazine construct to test the ability of this approach to fully biotinylate low affinity ligands *via* enzymatic turnover under sub-saturating conditions. A gel-shift assay was performed with a DNA-linked 110A ligand (Fig. 3d) with ∼10% bound to the target protein. With extended incubation (4 hours), the biotinylation of the DNA-linked 110A construct was nearly quantitative (Fig. 3e). As expected, the high affinity DNA-linked ligand BrBA was biotinylated more rapidly. Importantly, the biotinylation of non-ligand was not detectable, which shows promise for enrichment of weak affinity ligands while minimizing background signal. Under similar conditions, the 3′ amine modified-DNA nucleophile achieved only ∼50% biotinylation within 4 hours (Fig. S6†). Extended incubation under even lower fraction bound conditions (∼1%) were less successful presumably due to the Ultra-ID-target protein loosing its activity through self acylation. Further accumulation of a biotin label on constructs could potentially be achieved by replacement of the “exhausted” protein with fresh, active protein until background acylation of non-ligands becomes an issue.

These approaches do allow iterated selections to be performed through removal of the biotinylated strand by heat denaturation, which should allow even higher fold enrichments to be achieved in the presence of some background labelling. Iteration of traditional solid phase affinity selections is a common approach to increase enrichment of DEL ligands to detectable levels. We performed iterated selections against UltraID-CBX7-ChD at 2 μM using both a high-affinity ligand (BrBA, on-DNA *K*_d_ ≈ 50 nM) and a medium affinity peptide (Fig. S7,†*K*_d_ ≈ 2 μM).^28^ To elute DNA constructs in the first-round proximity-induced selection, the streptavidin beads were incubated with excess (100-fold) of the unmodified ssDNA-amine and heated above the *T*_m_ of the tethered 20-mer DNA duplex (68 °C) for 5 minutes. The recovered supernatant from the magnetic beads was used for a second-round (iterated) selection. qPCR analysis quantified the ligand enrichment for each round of the selection. As shown in Fig. S7,† the first-round selection yielded 5000-fold and 400-fold enrichment for BrBA and the medium affinity ligand, respectively. While the enrichment in the subsequent selection was lower than expected, the overall enrichment after two iterative selections reached approximately 0.5 million-fold for BrBA and 11 000-fold for the medium affinity ligand.

**Fig. 4:**
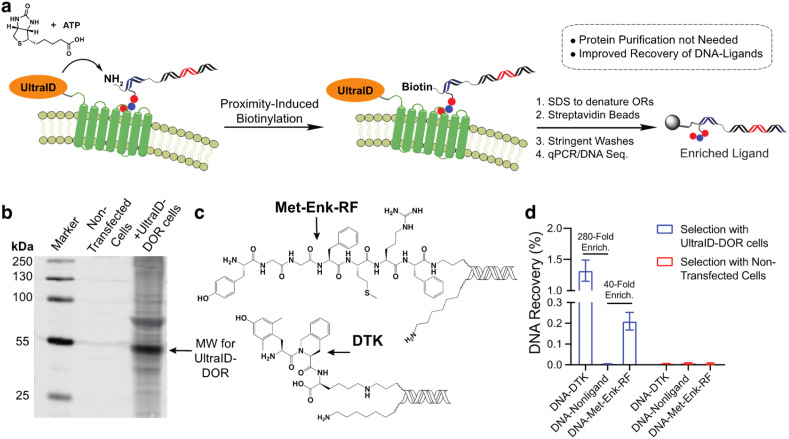
Proximity-induced biotinylation approach to live cell selections against membrane proteins. (a) Scheme for selection against UltraID-DOR on live cells. (b) Streptavidin blot for validation of the expression of UltraID-DOR and its biotinylation activity. Expi293F cells were transfected with a UltraID-DOR expression vector. After 24 hours expression, both transfected and untransfected cells with incubated with biotin (2 μM) and ATP (50 μM) for 30 minutes. Protein biotinylation was detected by LI-COR IRDye 800CW-streptavidin. (c) Structures of on-DNA DOR ligands Met-Enk-RF and DTK used in the selections. (d) qPCR analysis of the recovery of DNA constructs from test selections against UltraID-DOR. 24 hours post transfection, Expi293F cells with incubated with the 3 DNA constructs (0.1 nM, DTK-DNA, 0.1 nM Met-Enk-RF-DNA, 100 nM non-ligand DNA), biotin (2 μM), and ATP (50 μM). After 30 minutes, cells were washed and lysed with 1% SDS to denature proteins. Collected DNA was applied to streptavidin beads for purification, and the quantity of DNA constructs before and after purification was determined by qPCR.

In our previous work with a covalent crosslinking approach for DEL selections against membrane proteins on live cells, the recovery of high-affinity DNA-linked ligands from the selection was typically less than 0.1%.^15^ We attribute this to the difficulty in quantitative capture of the biotinylated membrane protein–DNA conjugate. As this proximity-induced biotinylation approach does not require this purification, we anticipated that this approach would improve the ligand recovery for live-cell selections against membrane proteins. We expressed an UltraID-δ opioid receptor fusion protein (UltraID-DOR) in Expi293F cells (ThermoFisher) and performed selections on live cells using two known ligands (Fig. 4c, Met-Enk-RF,^29^ ∼10 nanomolar binder to DOR; Dmt-Tic-Lys (DTK), sub-nanomolar binder to DOR).^15^ We first verified the expression and biotinylation activity of UltraID-DOR by treating cells with biotin and ATP for 10 min followed by a streptavidin blot to detect biotinylated proteins (Fig. 4b). Compared to cell lysates from non-transfected cells, biotinylation of cellular proteins was clearly observed. The molecular weight of the most intense band is consistent with the weight of autobiotinylated UltraID-DOR. We then performed a test selection against UltraID-DOR on live cells using the two DNA-encoded ligands and a non-ligand control (Fig. 4a). Following incubation, 1% SDS was added to denature membrane proteins to release tight binding ligands. Biotinylated DNA constructs were then captured by streptavidin beads. After stringent washing steps, the beads were boiled to elute DNA for qPCR analysis. The proximity-induced selection method achieved 280-fold and 40-fold enrichment for DTK and Met-Enk-RF, respectively. While this level of enrichment was similar to our previous report using crosslinking, the recovery of the ligand DNA was improved ∼10-fold (Fig. 4d).

## Conclusions

Enzyme-mediated proximity labelling approaches have expanded rapidly for mapping the interactome of proteins through genetic fusion to enzyme tags.^30^ For protein–nucleic acid interactions, an engineered ascorbate peroxidase (APEX) and a singlet oxygen-generating flavoprotein (MiniSOG) are the predominantly used tags. While these approaches could be used without further alteration of a DEL construct, labelling reactions by a phenoxyl radical (APEX) or oxidation and subsequent amine addition (MiniSOG) are destructive to both the target protein and the DNA. Given the minimal nature of DEL DNA constructs, such labelling could quickly lead to unamplifiable DNA. In extending like approaches to DEL assays, we took advantage of the ability to add functionality to the DNA in both the NLuc (addition of a photocaged biotin) and UltraID (addition of an amine/hydrazine) cases. This should minimize damage of either binding partner, particularly with the NLuc tags. This feature may enable other enzyme tags to be used for this purpose with further development. In the present work, we have used ssDNA tethered to encoding dsDNA constructs out of convenience, which facilitates the appending of various additional groups through DNA hybridization. Recent work from the Xiaoyu Li group has shown how exonuclease digestion can convert dsDNA DELs to ssDNA DELs.^31^ This will enable application of the presented approach to both commercially available “headpiece” dsDNA libraries as well as DELs prepared as ssDNA.^32^

The approach does require recombinant expression of a target protein with a fusion protein. Both NLuc and BioID variants have seen widespread use and are well tolerated. As fusion proteins go, these are fairly small (Nluc (19 kDa) and UltraID (20 kDa)). The potential modification of function for a target protein of interest would have to be assessed on a case-by-case basis. A potential advantage of the Nluc tag is that it can be expressed on a protein target as an 11 amino acid tag (HiBiT) that can reconstitute to the fully luminescent protein through high affinity binding (*K*_d_ = 700 pM) to the remainder of Nluc (LgBiT).^33^ The small HiBiT tag can be efficiently inserted into the genome as a tag on endogenous proteins using CRISPR/Cas9.

In summary, we developed a convenient and general approach for the selection of ligands from DNA-encoded libraries. This approach capitalizes on the power of enzyme activity to uncage or install a biotin purification tag on DNA-linked ligands and, in doing so, no protein purification or immobilization is needed in the selection step that assesses ligand binding to the target. Both Nluc- and UltraID-mediated methods effectively enrich DNA-linked ligands. A higher background was observed in Nluc-mediated selections. Improved photoprotecting groups for caging biotin would help minimize background signal and improve the selection efficiency. For the UltraID-based method, we demonstrated selections against both purified proteins and membrane protein targets on live cells, which improved recovery of ligands. We hope that the proximity-induced selection approach will serve as a useful tool for molecular discovery, particularly for the identification of low affinity ligands and for protein targets in complex biological samples.

## Data availability

More details can be found on the *Chemical Science* website (https://rsc.li/ChemSci_JSG).

## Author contributions

C. J. K. conceptualized the project. B. C. and C. J. K. designed experiments. B. C. synthesized all DNA conjugates and performed all *in vitro* and cellular assays. B. C. and C. J. K. synthesized coumarin-protected biotin 3. A. M. synthesized the hydrodabcyl quencher. B. C., A. M. and C. J. K. wrote the manuscript.

## Conflicts of interest

There are no conflicts to declare.

## Supplementary Material

SC-014-D2SC05495G-s001
