# Supplementary material for: Selection methods for proximity-dependent enrichment of ligands from DNA-encoded libraries using enzymatic fusion proteins
Source: Chem Sci. 2022 Nov 15;14(2):245–50. doi: 10.1039/d2sc05495g (PMC9811540; doi:10.1039/d2sc05495g)
Supplement: SC-014-D2SC05495G-s001 [file SC-014-D2SC05495G-s001.pdf]

Electronic Supplementary Material (ESI) for Chemical Science.
This journal is © The Royal Society of Chemistry 2022

Supplementary Information

Enzyme-mediated proximity labelling for enrichment of DNA-linked ligands from DNA-encoded libraries

Bo Cai, Amol Balasaheb Mhetre, Casey J. Krusemark*

Department of Medicinal Chemistry and Molecular Pharmacology, Purdue Center for Cancer Research, Purdue University, West Lafayette, IN 47907, USA

*Correspondence to: cjk@purdue.edu

Table of Contents

1. **Abbreviations S2**
2. **Supplementary Methods S3**
3. **Supplementary Schemes and Figures S14**
4. **Supplementary Spectra S21**
5. **Supplementary References S28**

1. Abbreviations

AcOH: Acetic acid

BSA: bovine serum albumin

DCM: dichloromethane

DIC: N,N'-diisopropylcarbodiimide

DEAE: diethylaminoethanol

DMA: N, N'-dimethylacetamide

DMF: N, N'-dimethylformamide

DMSO: dimethyl sulfoxide

DMAP: 4-dimethylaminopyridine

DOR: δ-opioid receptor

Dmt: 2,6-dimethyl-L-tyrosine

EDC: N-(3-dimethylaminopropyl)-N′-ethylcarbodiimide hydrochloride

Fmoc: 9-fluorenylmethyloxycarbonyl

HOAt: 1-Hydroxy-7-azabenzotriazole

HPLC: High performance liquid chromatography

IPTG: Isopropyl β-D-1-thiogalactopyranoside

Nluc: Nanoluc

NGS: next-generation sequencing

PBS: phosphate buffered saline

SDS-PAGE: sodium dodecyl sulfate-polyacrylamide gel electrophoresis

TEA: triethylamine

TFA: trifluoroacetic acid

TIC: 1,2,3,4-tetrahydroisoquinoline-3-carboxylic acid

TIPS: triisopropylsilane

1. Supplementary Methods

1.1 General Materials and Instrumentation

The organic solvents used in this work, including Dichloromethane (DCM), N, N-dimethylformamide (DMF), dimethyl sulfoxide (DMSO) and acetonitrile (CH_3CN) were purchased from Fisher Scientific. DNA oligonucleotides were obtained from Integrated DNA Technologies. Chemical reagents were purchased from commercial sources and used without further purification. Cell culture reagents were purchased from Thermo Fisher Scientific. Gibson Assembly Master Mix were purchased from New England Biolabs. DNA oligonucleotides and peptides were purified by reverse-phase high-pressure liquid chromatography (HPLC) on a Varian Pro Star HPLC system. Water in all reactions was filtered by a Millipore Milli-Q water filtration system. qPCR analysis of proximity-induced selections was performed on an Applied Biosystems™ ViiA™ 7 Real Time PCR system. Mass spectroscopy experiments were performed on a Waters Acquity UPLC with SQD2 mass spectrometer at the Purdue University Mass Spectrometry Center.

1.2 DNA Procedures

1.2.1 DNA Sequences and Modifications

ssDNAa'-3'-NH_2:
TGTGGCAAGCTTGATACCAT/3AmMO/
ssDNAa-linker-5'-NH_2:
/5AmMC12/ATGGTATCAAGCTTGCCACA
ssDNAa-linker-ssDNAb-5'-NH_2:
/5AmMC12/ATGGTATCAAGCTTGCCACA/iSp9/GTCGAGCTCTCTACTGCATA

1.2.2 Preparation of DNA-Linked Compounds

The peptidic ligands were synthesized on ssDNAa-linker-ssDNAb-5'-NH_2 using DEAE Sepharose (GE Healthcare, Catalog # 97067-950). The carboxylic acid derivative of coumarin caged biotin (obtained by hydrolyzing **Cou B** using porcine liver esterase[1], supplementary spectra S1) was conjugated to ssDNAa'-3'-NH_2. Briefly, 40 µL 50% DEAE slurry of DEAE Sepharose was pipetted into the well of a 384-well filter plates. The Sepharose was washed 3x with 50 µL H_2O, 2x with 50 µL DNA bind buffer (10 mM acetic acid with 0.005 % Triton X-100). 2 nmol of ssDNAa-linker-ssDNAb-5'-NH_2 was diluted in 180 µL DNA bind buffer. Diluted DNA was then applied to Sepharose in 3 aliquots of 60 µL each with 5-minute incubation. Following all additions,

Sepharose was washed 2x with 50 μL bind buffer, 2x with 50 μL H_2O and 2x with 50 μL MeOH. Each of the Fmoc-amino acids or the carboxylic acid derivative of coumarin caged biotin was coupled twice using standard HOAt/EDC conditions. Each coupling mixture contained 50 μL 25 mM HOAt in MeOH, 50 μL 150 mM Fmoc-AA stock in DMF and 25 μL 150 mM EDC-HCl in MeOH. 50 μL of the coupling reaction mixture was applied to Sepharose immediately and was allowed to incubate at room temperature for 30 minutes. Following each coupling, Fmoc deprotection was achieved by incubating sepharose with 50 μL 20% piperidine in DMF for 30 minutes. Finally, the sepharose was washed 3x with 50 μL DMF, 3x with 50 μL MeOH and 3x with 50 μL H_2O before elution using 3x 30 μL elution buffer (1.5 M NaCl, 0.005 % Triton X-100). DNA conjugates were purified by HPLC and confirmed on a Waters Acquity UPLC with SQD2 mass spectrometer.

1.2.3 Quantitative PCR (qPCR) Analysis

All qPCR reactions were prepared to a total volume of 10 μL and performed in 384-well plates (VWR, Catalog # 82051-464). Each 10 μL reaction contains 2.5 μL diluted DNA samples before/after selection assays, 2.5 μL 1 μM forward and reverse primers, and 5 μL Applied Biosystems™ PowerUp™ SYBR™ Green Master Mix (Fisher Scientific, Catalog # A25742). All qPCR reactions were performed in duplicates using the thermal cycling procedure described before.

1.3. Plasmid Construction, Protein Expression and Purification Procedures

1.3.1 Cloning

The Nluc plasmids were a gift from the Sjogren lab at Purdue University; Murine δ-opioid receptor (DOR) plasmid was a gift from Richard M. Van Rijn lab at Purdue University. UltraID plasmids were obtained from Addgene (Julien Béthune lab plasmids, plasmid ID 172879). The plasmids used in this work, including Nluc-CBX7-ChD, UltraID-CBX7-ChD and UltraID-DOR were constructed by Gibson Assembly. The sequences of all the plasmids used in this study were confirmed by Sanger Sequencing at Genewiz.

1.3.2 DNA Sequences of Proteins

Nluc-Linker-CBX7-ChD-6His:

ATGGTCTTCACACTCGAAGATTTCGTTGGGGACTGGCGACAGACAGCCGGCTACAAC
CTGGACCAAGTCCTTGAACAGGGAGGTGTGTCCAGTTTGTTTCAGAATCTCGGGGTG
TCCGTAACTCCGATCCAAAGGATTGTCCTGAGCGGTGAAAATGGGCTGAAGATCGA
CATCCATGTCATCATCCCGTATGAAGGTCTGAGCGGCGACCAAATGGGCCAGATCG
AAAAAATTTTTAAGGTGGTGTACCCTGTGGATGATCATCACTTTAAGGTGATCCTGC
ACTATGGCACACTGGTAATCGACGGGGTTACGCCGAACATGATCGACTATTTCGGAC
GGCCGTATGAAGGCATCGCCGTGTTCGACGGCAAAAAGATCACTGTAACAGGGACC
CTGTGGAACGGCAACAAAATTATCGACGAGCGCCTGATCAACCCCGACGGCTCCCT
GCTGTTCCGAGTAACCATCAACGGAGTGACCGGCTGGCGGCTGTGCGAACGCATTCT
GGCGTCTGGCGGTTCGGGAGGCGAGCAGGTGTTCGCCGTGGAGAGCATCCGGAAGA
AGCGCGTGCGGAAGGGTAAAGTCGAGTATCTGGTGAAGTGGAAAGGATGGCCCCCA
AAGTACAGCACGTGGGAGCCAGAAGAGCACATCTTGGACCCCCGCCTCGTCATGGC
CTACGAGGAGAAGGAGGAGTGA

UltraID-Linker-CBX7-ChD-6His:

ATGGAACAAAAACTCATCTCAGAAGAGGATCTCGACTTCAAGAACCTGATCTGGCT
GAAGGAGGTGGACAGCACCCAGGAGAGACTGAAGGAGTGGAACGTGTCCTACGGC
ACCGCCCTGGTGGCCGACAGACAGACCAAGGGCAGAGGCGGCCCGGGCAGAAAGT
GGCTGAGCCAGGAGGGCGGCCTGTACTTCAGCTTCCTGCTGAACCCCAAGGAGTTC
GAGAACCTGCTGCAGCTGCCCCTGGTGCTGGGCCTGAGCGTGAGCGAGGCCCTGGA
GGAGATCACCGAGATCCCCTTCAGCCTGAAGTGGCCCAACGACGTGTACTTCCAGG
AGAAGAAGGTGAGCGGCGTGCTGTGCGAGCTGAGCAAGGACAAGCTGATCGTGGGC
ATCGGCATCAACGTGAACCAGAGAGAGATCCCCGAGGAGATCAAGGACAGAGCCA
CCACCCTGTACGAGATCACCGGCAAGGACTGGGACAGAAAGGAGGTGCTGCTGAAG
GTGCTGAAGAGAATCAGCGAGAACCTGAAGAAGTTCAAGGAGAAGTCTGGCGGTTCG
GGAGGCGGGAGTGGAGGTGAGCAGGTGTTCGCCGTGGAGAGCATCCGGAAGAAGCGC
GTGCGGAAGGGTAAAGTCGAGTATCTGGTGAAGTGGAAAGGATGGCCCCCAAAGTA
CAGCACGTGGGAGCCAGAAGAGCACATCTTGGACCCCCGCCTCGTCATGGCCTACG
AGGAGAAGGAGGAGTGA

UltraID-Linker-DOR (murine):

ATGGAACAAAAACTCATCTCAGAAGAGGATCTCGACTTCAAGAACCTGATCTGGCT
GAAGGAGGTGGACAGCACCCAGGAGAGACTGAAGGAGTGGAACGTGTCCTACGGC
ACCGCCCTGGTGGCCGACAGACAGACCAAGGGCAGAGGCGGCCCGGGCAGAAAGT
GGCTGAGCCAGGAGGGCGGCCTGTACTTCAGCTTCCTGCTGAACCCCAAGGAGTTC
GAGAACCTGCTGCAGCTGCCCCTGGTGCTGGGCCTGAGCGTGAGCGAGGCCCTGGA
GGAGATCACCGAGATCCCCTTCAGCCTGAAGTGGCCCAACGACGTGTACTTCCAGG
AGAAGAAGGTGAGCGGCGTGCTGTGCGAGCTGAGCAAGGACAAGCTGATCGTGGGC
ATCGGCATCAACGTGAACCAGAGAGAGATCCCCGAGGAGATCAAGGACAGAGCCA
CCACCCTGTACGAGATCACCGGCAAGGACTGGGACAGAAAGGAGGTGCTGCTGAAG
GTGCTGAAGAGAATCAGCGAGAACCTGAAGAAGTTCAAGGAGAAGTCTGGCGGTTCG
GGAGGCGGGAGTGGAGGTATGGAGCTGGTGCCCTCTGCCCGTGCGGAGCTGCAGTCCT
CGCCCCTCGTCAACCTCTCGGACGCCTTTCCCAGCGCCTTCCCCAGCGCGGGCGCCA
ATGCGTCGGGGTCGCCGGGAGCCCGTAGTGCCTCGTCCCTCGCCCTAGCCATCGCCA
TCACCGCGCTCTACTCGGCTGTGTGCGCAGTGGGGCTTCTGGGCAACGTGCTCGTCA

TGTTTGGCATCGTCCGGTACACCAAATTGAAGACCGCCACCAACATCTACATCTTCA
ATCTGGCTTTGGCTGATGCGCTGGCCACCAGCACGCTGCCCTTCCAGAGCGCCAAGT
ACTTGATGGAAACGTGGCCGTTTGGCGAGCTGCTGTGCAAGGCTGTGCTCTCCATTG
ACTACTACAACATGTTCACTAGCATCTTCACCCTCACCATGATGAGCGTGGACCGCT
ACATTGCTGTCTGCCATCCTGTCAAAGCCCTGGACTTCCGGACACCAGCCAAGGCCA
AGCTGATCAATATATGCATCTGGGTCTTGGCTTCAGGTGTCGGGGTCCCCATCATGG
TCATGGCAGTGACCCAACCCCGGGATGGTGCAGTGGTATGCATGCTCCAGTTCCCCA
GTCCCAGCTGGTACTGGGACACTGTGACCAAGATCTGCGTGTTCCTCTTTGCCTTCGT
GGTGCCGATCCTCATCATCACGGTGTGCTATGGCCTCATGCTACTGCGCCTGCGCAG
CGTGCGTCTGCTGTCCGGTTCCAAGGAGAAGGACCGCAGCCTGCGGCGCATCACGC
GCATGGTGCTGGTGGTGGTGGGCGCCTTCGTGGTGTGCTGGGCGCCCATCCACATCT
TCGTCATCGTCTGGACGCTGGTGGACATCAATCGGCGCGACCCACTTGTGGTGGCCG
CACTGCACCTGTGCATTGCGCTGGGCTACGCCAACAGCAGCCTCAACCCGGTTCTCT
ACGCCTTCCTGGACGAGAACTTCAAGCGCTGCTTCCGCCAGCTCTGTCGCACGCCCT
GCGGCCGCCAAGAACCCGGCAGTCTCCGTCGTCCCCGCCAGGCCACCACGCGTGAG
CGTGTCACTGCCTGCACCCCCTCCGACGGCCCGGGCGGTGGCGCTGCCGCCTGA

1.3.3. Protein Expression and Purification

The Nluc-CBX7-ChD/UltraID-CBX7-ChD with His-tag in pet28b (+) expression vector was transformed into *E. coli* BL21 (DE3) Rosetta cells (Novagen®, purchased from Fisher Scientific, Catalog # 70-956-3). Approximately 25 µL of each transformation was plated onto a 10 cm LB agar plates with antibiotics (chloramphenicol (34 µg/mL) and kanamycin (50 µg/mL)). The plates were allowed to incubate at 37°C overnight. On the next day, a single fresh colony was selected and shaken at 225 rpm in 3 mL of LB broth with chloramphenicol and kanamycin (final concentration at 34 µg/mL and 50 µg/mL, respectively), After shaking at 37°C for 12 hours, 1 mL of the starter culture was added to 500 mL of LB broth with chloramphenicol and kanamycin (final concentration at 34 µg/mL and 50 µg/mL, respectively). The mixture was allowed to shake at 225 rpm at 37 °C until the optical density at 600 nm reached to 0.5. Isopropyl β-D-1-thiogalactopyranoside (IPTG) was added to the culture with a final concentration of 0.25 mM, followed by incubation at 22 °C for 18 h. Protein expression was confirmed by SDS-PAGE analysis. Protein purification was purified using HisPur™ Ni-NTA Magnetic Beads (Thermo Fisher Scientific, Catalog # 88831) following the manufacturer's protocol (supplementary Figure S1).

1.4. Synthetic Procedures

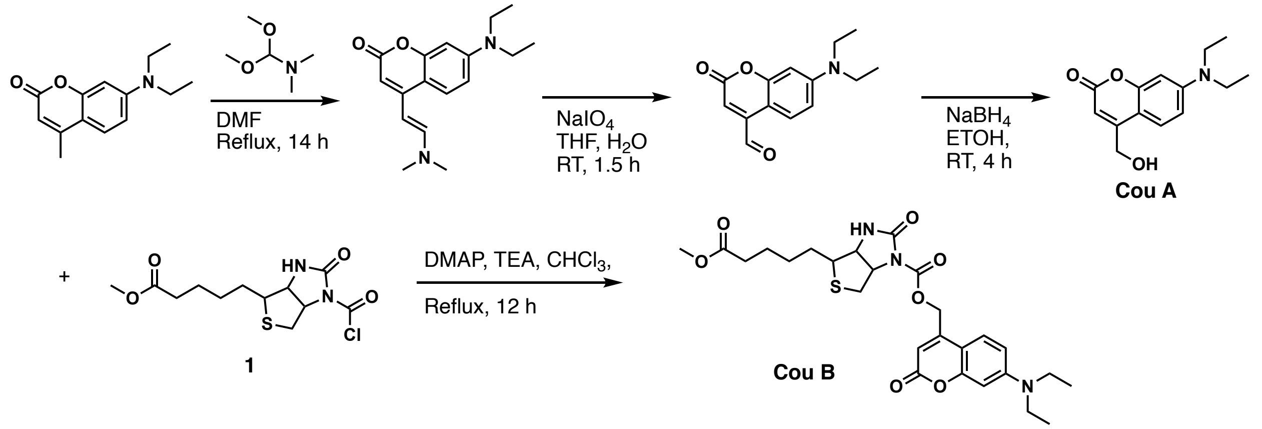

Scheme S1. Synthesis of photocaged biotin using a coumarin protecting group.

Cou B

The synthesis of compound **1** was performed following the protocol by Chen et al.[1] Briefly, to biotin methyl ester (200 mg, 0.77 mmol) was added triphosgene (0.68 g, 2.3 mmol) in 10 mL dry chloroform. The mixture was refluxed for 12 hours. The reaction was cooled, and the solvent was evaporated. The crude mixture was purified by flash column chromatography (50% EtOAc/50% hexanes) to yield compound **1 (**148 mg, 0.46 mmol, 60% yield). The synthesis of **Cou A** (7-diethylamino-4-hydroxymethyl coumarin) was performed according to the procedure described by Göbel et al.[2] To synthesize coumarin caged biotin **Cou B**, to a solution of **Cou A** (81 mg, 0.33 mmol) in 10 mL of dry $CHCl_3$ (protected from light using aluminum foil) was added compound **1** (35 mg, 0.11 mmol), 4-dimethylaminopyridine (DMAP, 40 mg, 0.33 mmol) and triethylamine (TEA, 46 µL, 0.33 mmol). The mixture was stirred under reflux for 12 hours. The reaction was cooled, and the solvent was evaporated. The crude mixture was purified by flash column chromatography (20% EtOAc in hexanes to 100% EtOAc) to yield **Cou B (**18.4 mg, 0.034 mmol, 10% yield).

^{1}H NMR (500 MHz, $CDCl_3$) δ 7.35 (d, J = 8.9 Hz, 1H), 6.61 (d, J = 9.4 Hz, 1H), 6.53 (d, J = 2.6 Hz, 1H), 6.27 (s, 1H), 5.45 – 5.32 (m, 2H), 4.89 (d, J = 6.5 Hz, 1H), 4.27 (s, 1H), 3.68 (s, 3H), 3.42 (q, J = 7.1 Hz, 4H), 3.22 (s, 1H), 3.13 (d, J = 13.6 Hz, 1H), 3.05 (dd, J = 13.7, 5.3 Hz, 1H), 2.35 (t, J = 7.3 Hz, 2H), 1.83 – 1.57 (m, 5H), 1.48 (s, 2H), 1.21 (t, J = 7.1 Hz, 6H).

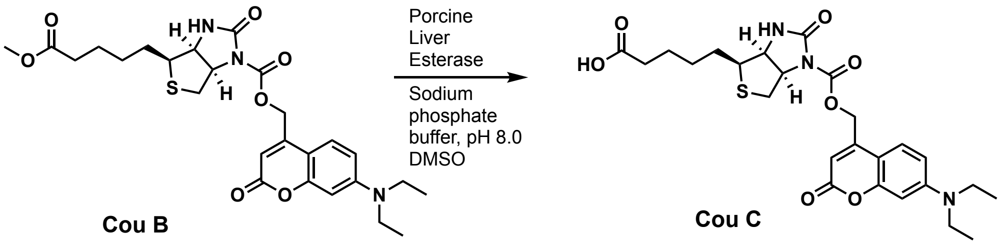

Scheme S2. Synthesis of the carboxylic acid derivative of coumarin-caged biotin.

Cou C

The synthesis of the carboxylic acid derivative of coumarin caged biotin (**Cou C**) was performed according to the procedure described by Terai et al.[3] To compound **Cou B** (10 mg, 0.019 mmol) was added 2 mL of 0.1 M sodium phosphate buffer (pH 8.0), 0.4 mL of DMSO, and 300 units of porcine liver esterase (Sigma-Aldrich). The mixture was allowed to stir at 200 rpm overnight at 32˚C. Desired product was extracted with 1 N HCl and ethyl acetate, dried over $MgSO_4$ and concentrated under reduced pressure. The mixture was purified by reverse-phase HPLC on an Agilent 1100 Series HPLC system using a H_2O/MeCN + 0.1 % TFA gradient to yield **Cou C** (2.6 mg, 0.005 mmol, 26% yield). The purity and mass of **Cou C** were confirmed by LC-MS (Supplementary Spectra). LCMS-ESI: *m/z* Calcd for $C_{25}H_{31}N_3O_7S$ $(M+ H)^+$, 518.19; found: 518.30.

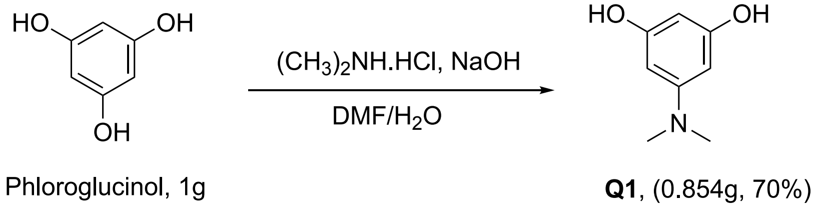

Scheme S3. Synthesis of 5-(dimethylamino)benzene-1,3-diol (**Q1**)

5-(dimethylamino)benzene-1,3-diol (Q1)

The 5-(dimethylamino)benzene-1,3-diol was synthesized according to the procedure described by Kempf et al.[5] from phloroglucinol (1 g, 7.929 mmol). Compound **Q1** was obtained as pink crystal solid after purification by silica column chromatography using 50% ethyl acetate in hexanes as an eluent (0.854 g, 70%); ^{1}HNMR (500 MHz, DMSO-d_6): $\delta = 8.83$ (s, 2H), 5.59 (s, 3H), 2.77 (s, 6H). LCMS-ESI: *m/z* Calcd for $C_8H_{12}NO_2$ $(M + H)^+$, 154.09; found: 154.10.

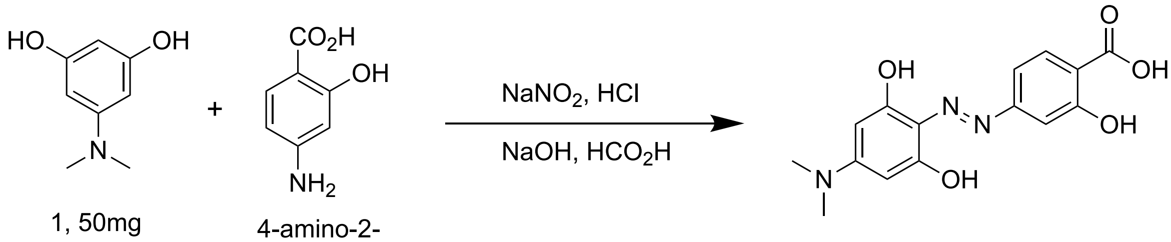

Scheme S4. Synthesis of 5-(dimethylamino)benzene-1,3-diol (**Q2**)

(E)-4-((4-(dimethylamino)-2,6-dihydroxyphenyl)diazenyl)-2-hydroxybenzoic acid (Q2)

The quencher (**Q2**) was synthesized using a previously published methodology from Kempf et al.[5] with minor modifications. To a cold solution of 4-aminosalicylic acid (50 mg, 0.326 mmol) in 6 M HCl (172 µL), ice cold 2.5 M solution of $NaNO_2$ (260 µL) was added. The mixture was stirred for 1 h at 0 °C and then added dropwise to a solution of compound 1 (50 mg, 0.326 mmol) in 1M aq. NaOH (680 uL) at 0 °C. The reaction mixture was heated at 75 °C for 30 min and then at RT for 2h. The reaction mixture diluted with MeOH (4 mL). The mixture was filtered and then filtrate concentrated under vacuum. The concentrated residue diluted with 0.1 M sodium hydroxide solution (3 mL), filtered, and the filtrate was treated with formic acid and ethanol (2 mL) adjusting a pH < 4.0. The mixture was placed at 4 °C for 16 h and then centrifuged for 10 min at 4 °C (Allegra X-15 R Centrifuge operating at 3000 rpm). The pellet obtained was dispersed in 0.1 % formic acid (5 mL) and again centrifuged as before; this procedure was repeated 3 times. Then the residue was washed with distilled water (2x5 mL) and centrifuged; finally, the product was re-suspended in distilled water (2 mL) and dried by lyophilisation to obtain the quencher **Q2** as a red powder (34 mg, 33%). The compound containing glass vial was covered with aluminium foil and stored at 4 °C. ^{1}H NMR (500 MHz, DMSO-d_6): δ = 7.79 (d, J = 8.5 Hz, 1H), 7.41 (d, J = 1.8 Hz, 1H), 7.19 (dd, J = 8.5, 1.8 Hz, 1H), 5.71 (s, 2H), 3.08 (s, 6H); LCMS (ESI) *m/z* Calcd for $C_{15}H_{16}N_3O_5$ $(M + H)^+$, 318.11; found: 318.20.

Synthesis of Met-Enk-RF, weak affinity ligand 110A and Dmt-Tic-Lys (DTK)

The alkyne derivative of Met-Enk-RF and 110A was synthesized on (FMPB AM resin, Novabiochem®, Catalog # 8550280005) as previously described.[3] Briefly, 100 mg of FMPB AM resin was added to a round bottom flask and stirred in DCM at room temperature for 20 minutes. The DCM was removed by gentle evaporation, and 5 mL of MeOH with 5 mL of DMF was added

to the resin. To the mixture was then added 10 eq. glacial AcOH, 10 eq. propargyl amine and 10 eq. $NaCNBH_3$. The mixture was then gently stirred under light reflux for 3 hours at 80 °C. The mixture was cooled, washed with DMF, MeOH and DCM, and then re-swelled in 1,2-dichloroethane prior to the first acylation with Fmoc-amino acids. After the removal of 1,2-dichloroethane, each Fmoc-protected amino acid was then coupled to the resin using 5 eq. Fmoc-amino acid, 5 eq. HOAt, 5 eq. DIC in DMF. For each coupling, the Fmoc-amino acid, HOAt, DIC mixture was pre-activated for 20 minutes at room temperature before being added to the resin. Each coupling was allowed to proceed at room temperature for 60 minutes. Fmoc deprotection was completed by incubating the resin in 20% piperidine in DMF for 30 minutes. Finally, the peptide was cleaved by incubating in 95 % TFA, 2.5 % triisopropylsilane, and 2.5 % H2O for 3 hours. The crude peptide was precipitated out of ice-cold diethyl ether and purified by HPLC using a H_2O/MeCN + 0.1 % TFA gradient with detection at 215 nm. The Dmt-Tic-Lys (2,6-dimethyl-L-tyrosine (Dmt), 1,2,3,4-tetrahydroiso-quinolone-3-carboxylic acid (Tic)) was synthesized on Wang resin (Novabiochem®) as previously described. The mass and purity of the peptides were confirmed by the Waters Acquity UPLC with SQD2 mass spectrometer at Purdue Mass Spectrometry Center.

Enzyme-Mediated Proximity Labelling Selection Assay
Proximity-Induced Photo-Deprotection Approach to DEL selection

A premix of DNA-encoded BrBA and non-ligand-DNA (final concentration 0.1 nM and 100 nM, respectively) was added to 2 μM Nluc-CBX7-ChD in 100 mM sodium phosphate buffer, pH 8.0, 150 mM NaCl, 0.02% (v/v) Tween-20 with 1 mg/mL BSA and 1 mg/mL Salmon Sperm DNA (Thermo Fisher Scientific). ssDNAa'-3'-caged biotin DNA was incubated in dark with 1 μL pre-washed NanoLink® Streptavidin Magnetic Beads (Solulink, Catalog # M-1002-010) for 30 minutes to remove any biotin-DNA that results from hydrolysis. The supernatant containing caged biotin-DNA was then added to the mixture (final concentration of caged biotin DNA = 200 nM). Furimazine was added to a final concentration of 50 μM. The mixture was covered in aluminum foil and incubated on a rotator at room temperature for 30 minutes. Following incubation, the mixture was incubated with 1 μL pre-washed NanoLink® Streptavidin Magnetic Beads (Solulink, Catalog # M-1002-010) at room temperature for 30 minutes. After incubation, the supernatant was removed, and beads were washed three times with PBS in the presence of 1 mg/mL BSA, 1 mg/mL Salmon Sperm DNA and 0.2% SDS. For the fourth and fifth wash, beads were washed with PBS.

After the final wash, beads were re-suspended in 20 μL pure water. DNA constructs were eluted by heating at 95 °C for 5 minutes. qPCR analysis of the premix samples before and after selection was performed to quantify the DNA recovery and enrichment factors.

Proximity-Induced Biotinylation Approach to DEL Selection

For selections with UltraID-CBX7-ChD, a premix of DNA-encoded ligand and non-ligand-DNA (final concentration 0.1 nM and 100 nM, respectively) was added to 2 μM UltraID-CBX7-ChD in 100 mM sodium phosphate buffer, pH 7.4, 5 mM $MgCl_2$, 150 mM NaCl, 0.02% (v/v) Tween-20 with 1 mg/mL BSA and 1 mg/mL Salmon Sperm DNA (Thermo Fisher Scientific). Following a 15-minute incubation, ssDNAa'-3'-free amine, biotin, ATP were added to the mixture to a final concentration of 200 nM, 5 μM and 2.5 mM, respectively. The mixture was allowed to incubate on a rotator at room temperature for 30 minutes. Following incubation, the mixture was incubated with 1 μL pre-washed NanoLink® Streptavidin Magnetic Beads (Solulink, Catalog # M-1002-010) at 37°C for 1 hour (note: before the streptavidin pulldown experiment, the DNA mixture can first be purified to remove the free biotin in solution). After incubation, the supernatant was removed, and beads were washed three times with PBS in the presence of 1 mg/mL BSA, 1 mg/mL Salmon Sperm DNA and 0.2% SDS. For the fourth and fifth wash, beads were washed with PBS. After the final wash, beads were re-suspended in 20 μL pure water. DNA constructs were eluted by heating at 95 °C for 5 minutes. qPCR analysis of the premix samples before and after selection was performed to quantify the DNA recovery and enrichment factors. For UltraID-DOR, approximately 2.5 million Expi293F™ cells expressing UltraID-DOR were harvested, washed twice with PBS and resuspended in 50 μL PBS. A premix of DNA-encoded DTK, Met-Enk-RF and non-ligand-DNA (final concentration 0.1 nM, 0.1 nM and 100 nM, respectively) was added to cells. Following a 15-minute incubation, ssDNAa'-3'-free amine, biotin, ATP, $MgCl_2$ were added to a final concentration of 200 nM, 2 μM, 2.5 mM and 5 mM, respectively. The mixture was allowed to incubate on a rotator at 37°C for 30 minutes. Follwing incubation, 1% SDS was added to denature membrane proteins to release tight binding ligands. Supernatant was recovered by centrifuging at 16,000 x g for 25 minutes. The supernatant was diluted with 4x PBS, streptavidin pulldown experiment and qPCR analysis were performed as described above.

Cell Culture

Expi293F™ suspension cells were obtained from Thermo Fisher Scientific (Catalog # A14527). Cells were cultured in 25 mL Erlenmeyer flasks with 4 mL of Expi293™ Expression Medium. Flasks were shaken at 130 rpm using an orbital shaker (KJ-201 BD) placed in a 37°C incubator with 8% CO_2 and > 85% relative humidity. For proximity-induced selection on live cells, UltraID-DOR plasmid in pcDNA 3.1(+) vector was transfected using ExpiFectamine™ 293 Reagent based on the manufacturer's protocols.

Imaging Experiment Procedures

Gel-Shift Assays

The gel-shift assays were performed to detect the presence of biotinylated DNA constructs. For Nluc-CBX7-ChD, 1 μM DNA-linked BrBA was incubated with 2 μM Nluc-CBX7-ChD for 15 minutes. ssDNAa'-3'-caged biotin DNA was incubated in dark with 1 μL pre-washed NanoLink® Streptavidin Magnetic Beads (Solulink, Catalog # M-1002-010) for 30 minutes to remove any biotin-DNA that results from hydrolysis. The supernatant containing caged biotin-DNA was then added to the mixture (final concentration of caged biotin DNA = 1 μM). Furimazine was added to the mixture to a final concentration of 50 μM. The mixture was covered in aluminum foil to protect from ambient light. The positive control sample was prepared by directly shining UV light on caged biotin DNA at 365 nm for 10 minutes. Negative control samples using non-ligand DNA, non-caged biotin construct, no protein or no furimazine were included. Following a 30-minute incubation, DNAs were incubated with 2 μg Neutravidin (deglycosylated avidin, Fisher Scientific, Catalog # PI31000) in PBS for 15 minutes and run on a 3% agarose gel. For UltraID-CBX7-ChD, 1 μM DNA-linked BrBA and DNA-linked free amine/hydrazine were incubated with UltraID-CBX7-ChD in PBS (pH 7.4) at indicated concentration in the presence of 50 μM biotin, 2.5 mM ATP and 5 mM $MgCl_2$. Following incubation, DNAs were purified by ethanol precipitation, incubated with 10 μg Neutravidin in PBS and run on a 10% TBE gel. Gels were stained with GelStar™ Nucleic Acid Gel Stain (Lonza, Catalog # 50535) and scanned using azure biosystems sapphire biomolecular imager. For tests performed under sub-saturating conditions, 1 μM DNA-linked 110A was incubated with 1 μM enzyme (Nluc/UltraID-CBX7-ChD). The rest steps of the gel shift assays were performed using the same protocol as described above.

In-Gel Streptavidin Blot

Non-transfected Expi293F^{TM} cells (Thermo Fisher Scientific, Catalog # A14635) and cells expressing UltraID-DOR were incubated with 2 μM biotin and 50 μM ATP in PBS at 37°C. After a 30-minute incubation on a rotator, cells were washed with PBS twice and lysed directly using SDS-PAGE buffer. Following a brief sonication of the samples, samples were loaded on an SDS-PAGE gel. Proteins were separated by electrophoresis at 150 V for 60 minutes. Following electrophoresis, the stacking gel was removed, and the remaining gel was gently shaked in fixing buffer (50% isopropanol + 5% acetic acid solution) for 15 minutes. The gel was then washed with Milli-Q water for 30 min, and incubated in PBS in the presence of 0.1% Tween 20, 0.05% SDS and 1% BSA. After incubation at room temperature for 1 h, LI-COR IRDye 800CW-Streptavidin (Fisher Scientific, Catalog # NC9386176, dilute at 1:5000) was added and the mixture was allowed to shake for another 60 minutes at room temperature. Finally, the gel was washed twice in PBS with 0.1% Tween 20 and 0.05% SDS. Gels were scanned using an Odyssey® Imaging System.

Supplementary Schemes and Figures

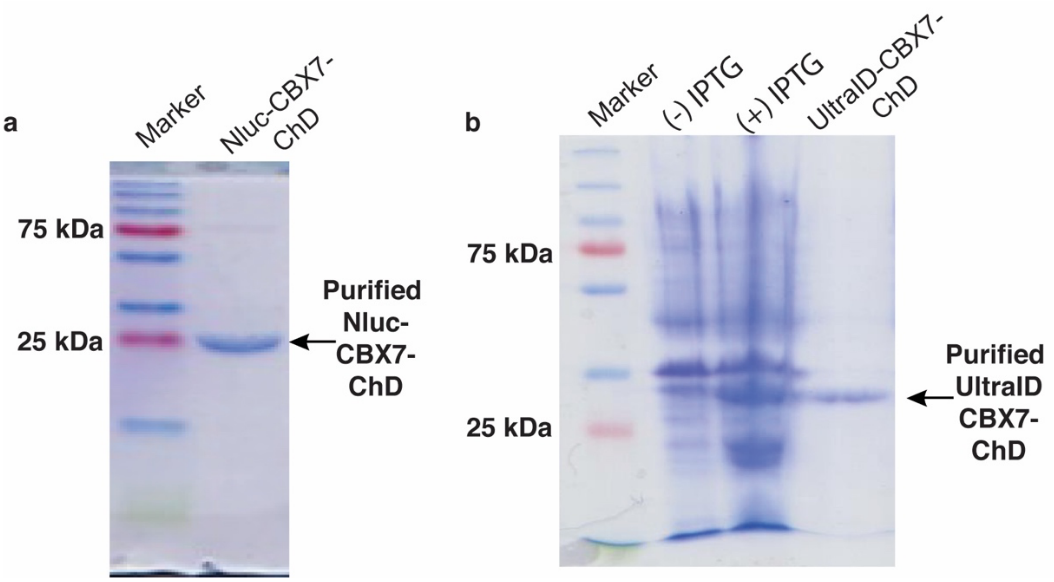

Figure S1. Purity of recombinant protein targets. SDS-PAGE analysis of Ni-NTA purified (a) Nluc-CBX7-ChD-6His and (b) UltraID-CBX7-ChD-6His. Expected molecular weight for Nluc-CBX7-ChD-6His: ≈ 24 kDa; UltraID-CBX7-ChD-6His: ≈ 29 kDa.

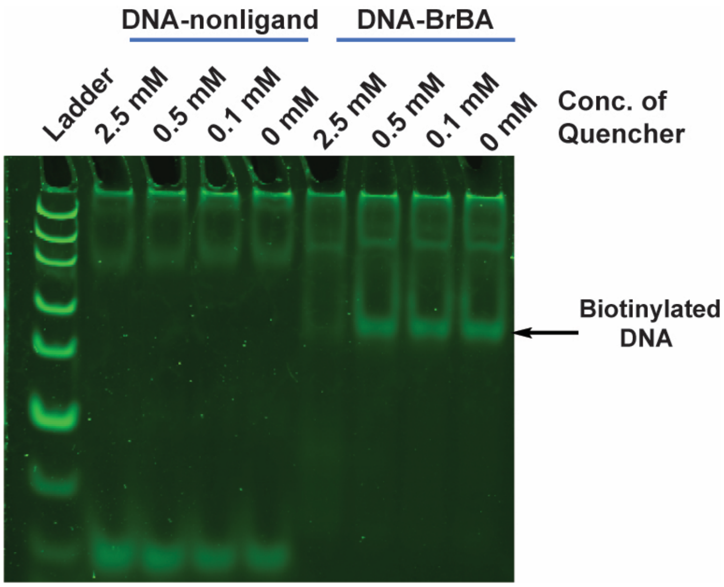

Figure S2. Assessment of the effect of the hydrodabcyl quencher on desired proximity-based deprotection. DNA-linked nonligand or BrBA (1 μM) was incubated with Nluc-CBX7-ChD (2 μM), furimazine (50 μM) and varying concentrations of the hydrodabcyl quencher for 30 minutes. Prior to PAGE analysis, excess Neutravidin (2 μM) was added.

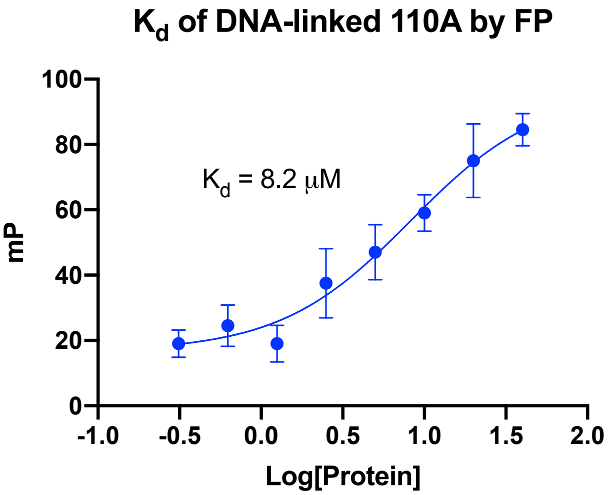

Figure S3. Analysis of DNA-110A ligand binding to CBX7-ChD by direct fluorescence polarization assay. 100 nM of FAM-conjugated DNA-110A ligand was incubated with increasing concentrations of recombinant CBX7-ChD. Curves were fit using "One site - Total" mode in GraphPad Prism 7.

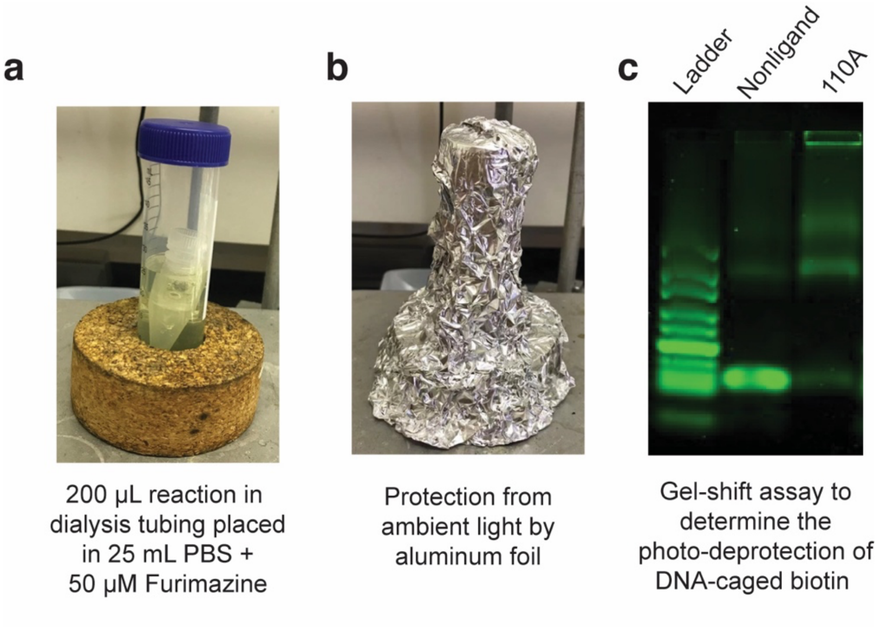

Figure S4. Nluc-mediated photo-deprotection of caged biotin with weak-affinity ligands in a dialysis chamber. (a) Experimental setup. A small volume dialysis chamber (Pur-A-Lyzer Midi, MWCO 3500 Da, Sigma Aldrich) contained a total sample volume of x µL with 1 µM each DNA construct (ligand and non-ligand, 50 pmol each), 1 µM Nluc-CBX7-ChD (50 pmol total) and 50 µM furimazine in PBS. The dialysis tube was placed in a stirred tube containing 25 mL of PBS and 50 µM furimazine (1.3 µmol total). (b) The reaction mixture was protected from ambient light to minimize background photo-deprotection. (c) Gel shift assay for studying the BRET-induced uncaging of coumarin-protected biotin after 6 hours. Prior to gel analysis, DNAs were incubated with 2 µg Neutravidin in PBS.

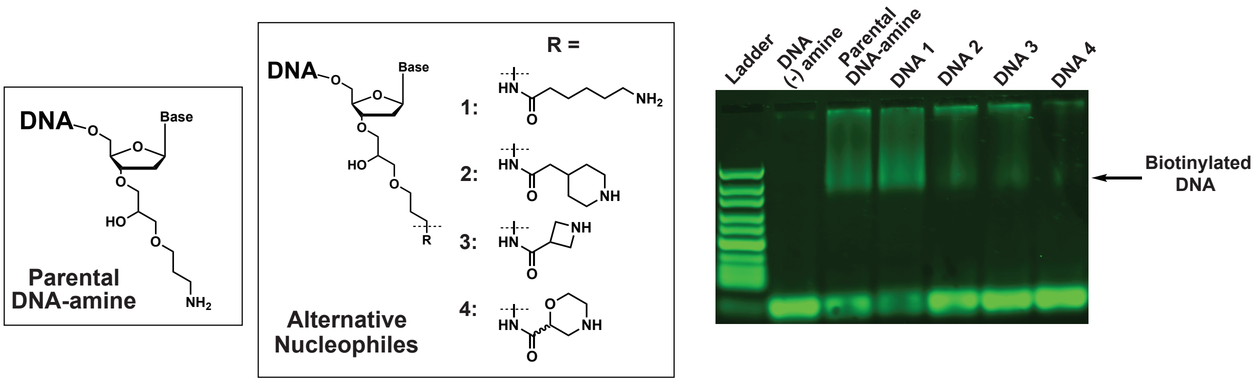

Figure S5. Assessment of the effect of amine linker length and alternative nucleophiles on UltraID-induced biotinylation efficiency. DNA-linked 110A (1 µM) was incubated with UltraID-CBX7-ChD (2 µM) and different DNA-linked nucleophiles in the presence of biotin (50 µM), ATP (2.5 mM) and $MgCl_2$ (5 mM) for 30 minutes. Prior to PAGE analysis, DNA was purified from the excess biotin by precipitation and then incubated with Neutravidin (10 µM).

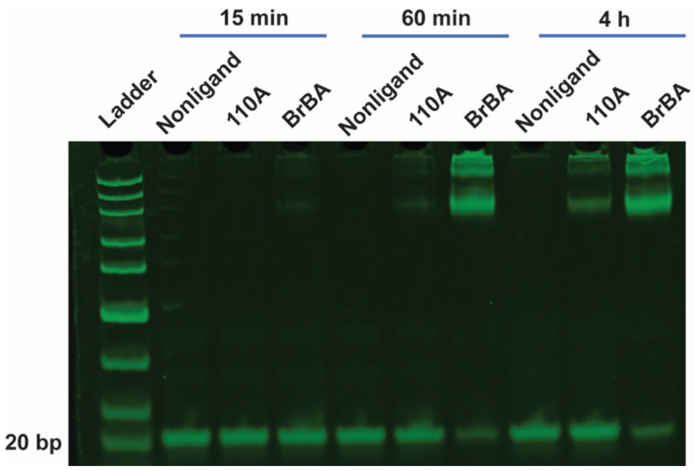

Figure S6. Gel shift assay for studying the UltraID-induced biotinylation of DNA-linked BrBA/110A with 3' primary amine modified-DNA nucleophile. DNA constructs were treated as in Fig.3b and incubated for the indicated times. Prior to gel analysis, DNAs were purified by ethanol precipitation and incubated with 10 μg Neutravidin in PBS.

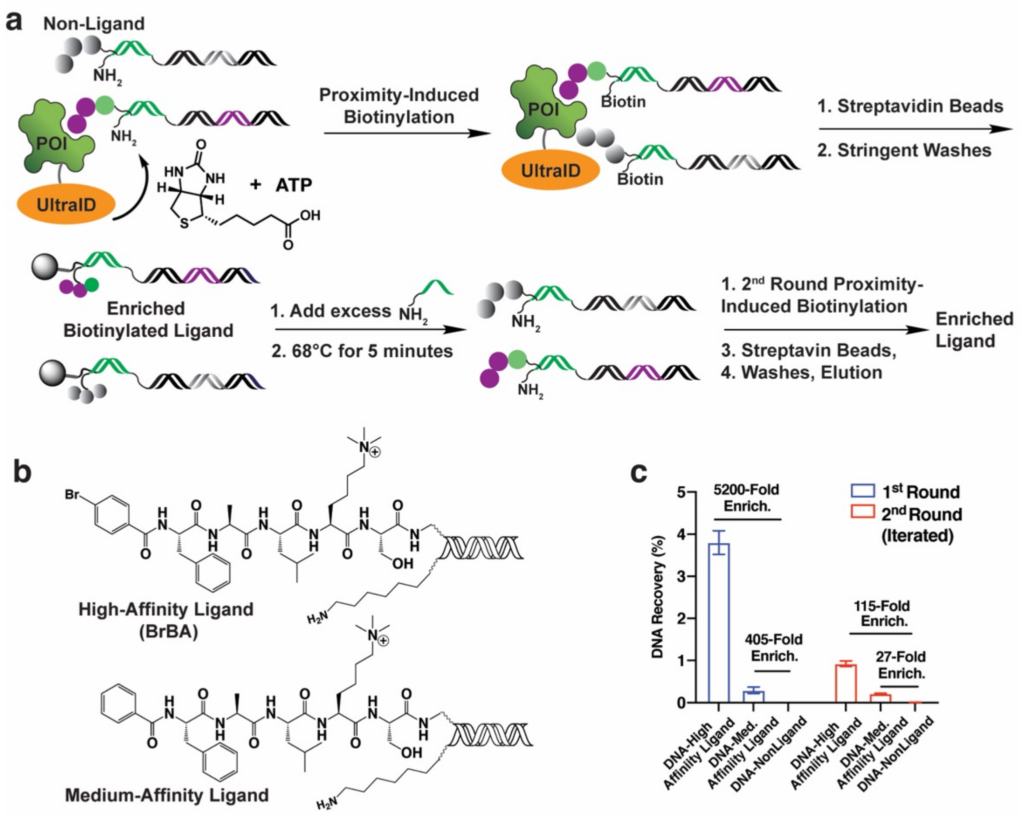

Figure S7. Proximity-induced biotinylation approach to DEL selection. (a) Scheme of iterated selection with UltraID-CBX7-ChD. (b) Structures of high-affinity and medium-affinity ligands used in selection. (c) qPCR analysis of DNA recovery from iterated test selections against UltraID-CBX7-ChD.

Supplementary Spectra

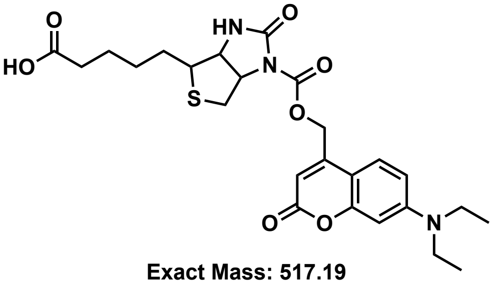

Exact Mass: 517.19

Purity confirmation of Cou C by LC/MS:

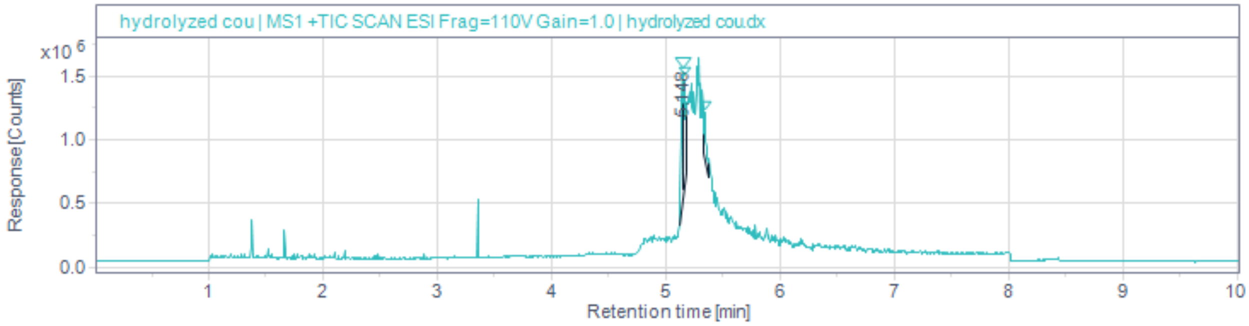

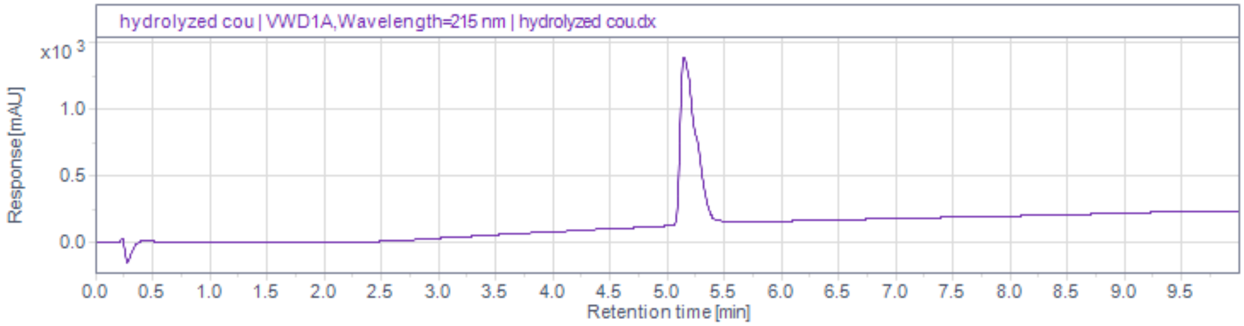

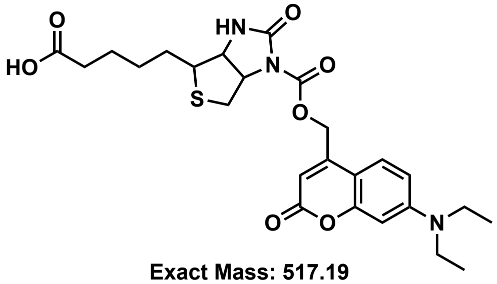

Exact Mass: 517.19

Mass confirmation of Cou C:

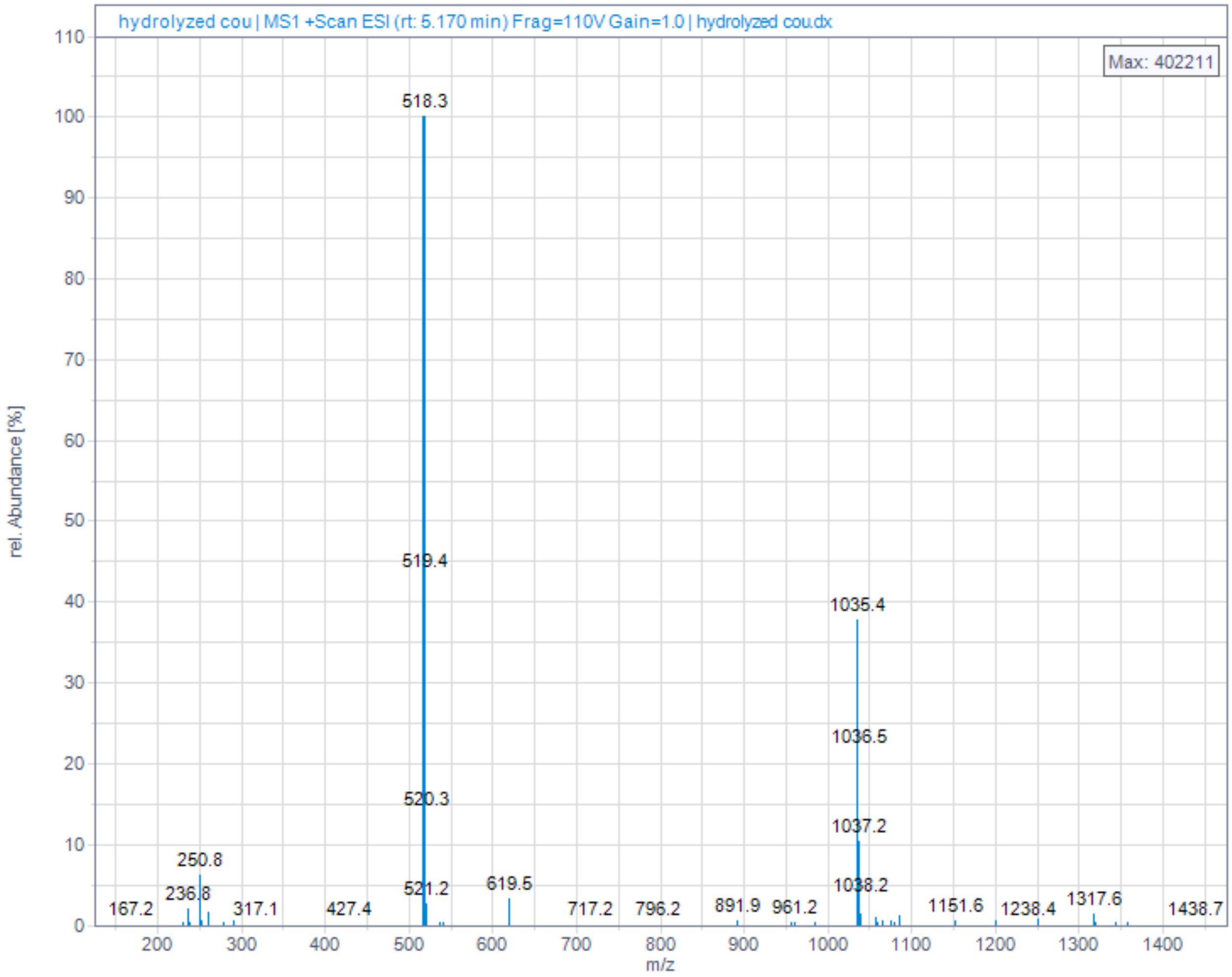

Purity confirmation of Q2:

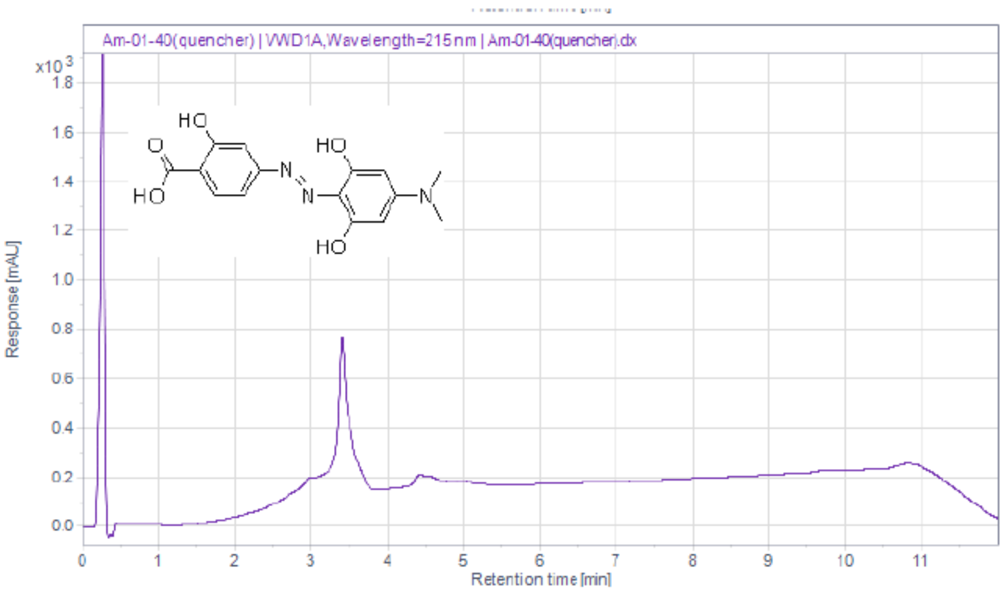

Mass confirmation of Q2:

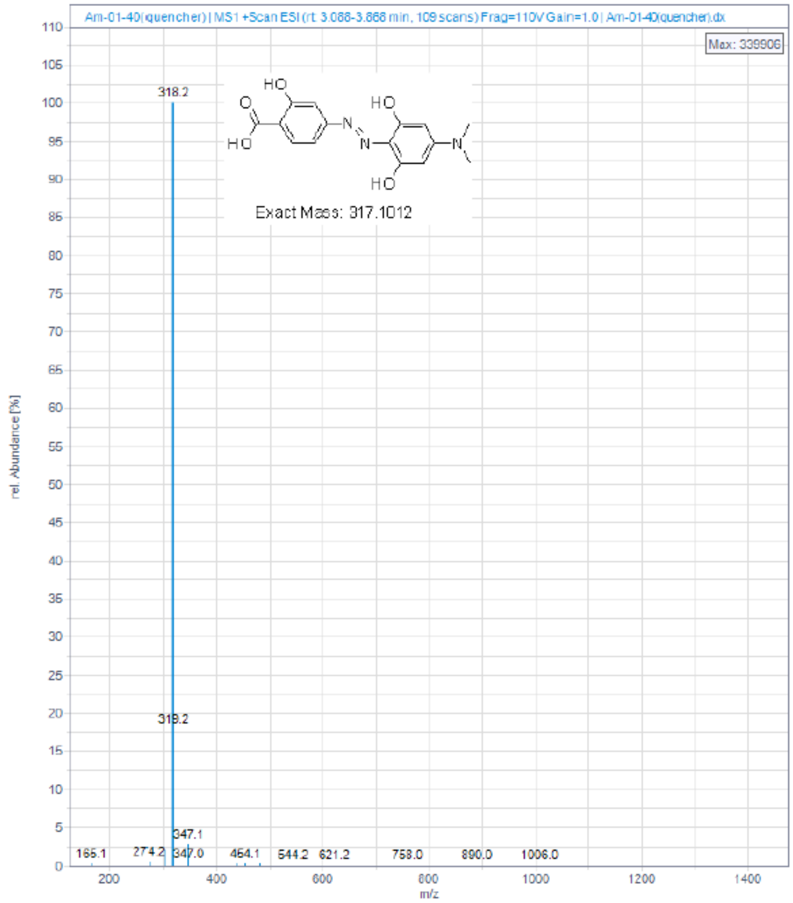

^{1}H NMR spectrum of coumarin caged biotin:

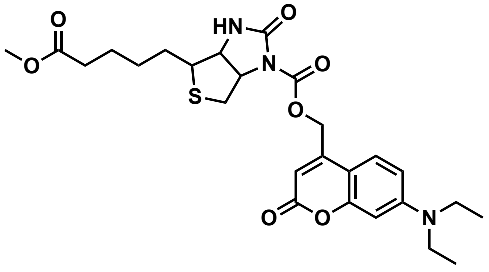

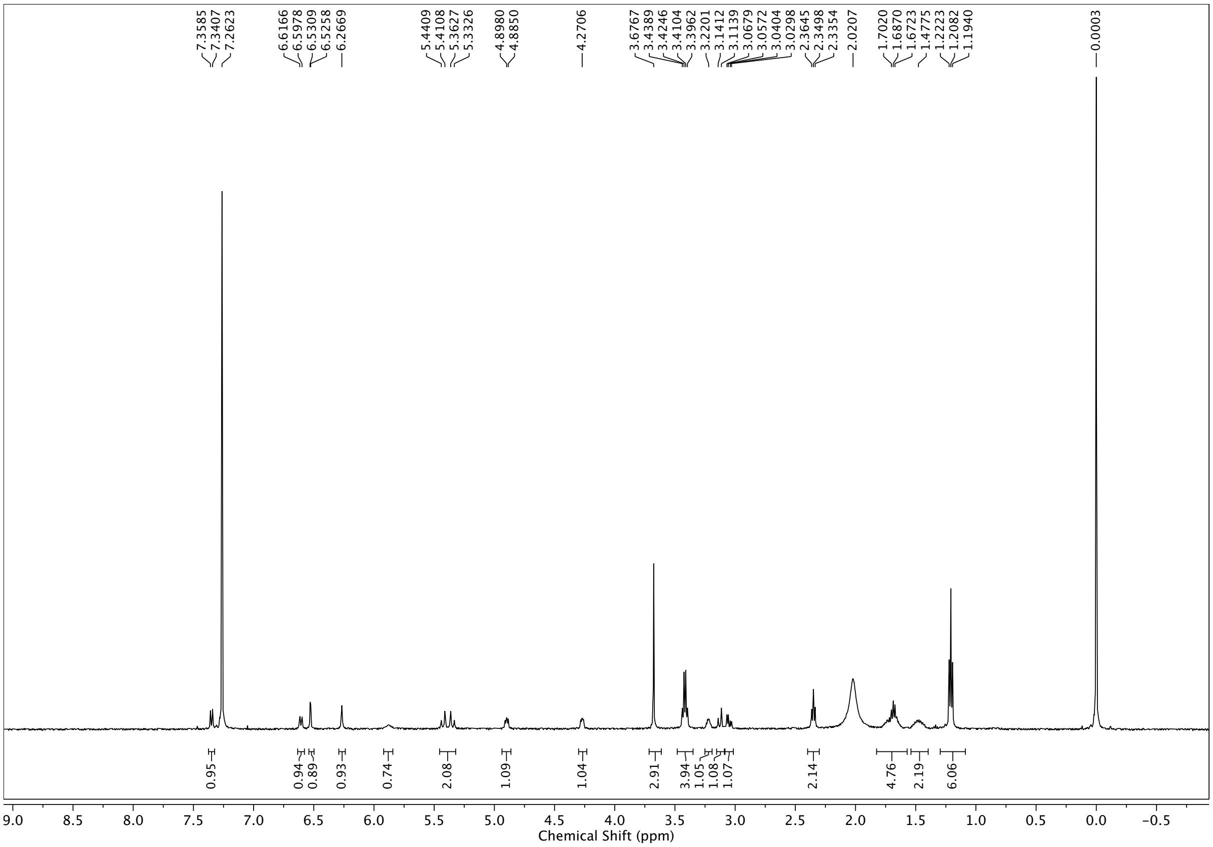

^{1}H NMR spectrum of Q1:

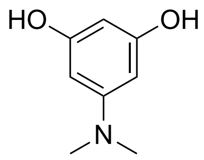

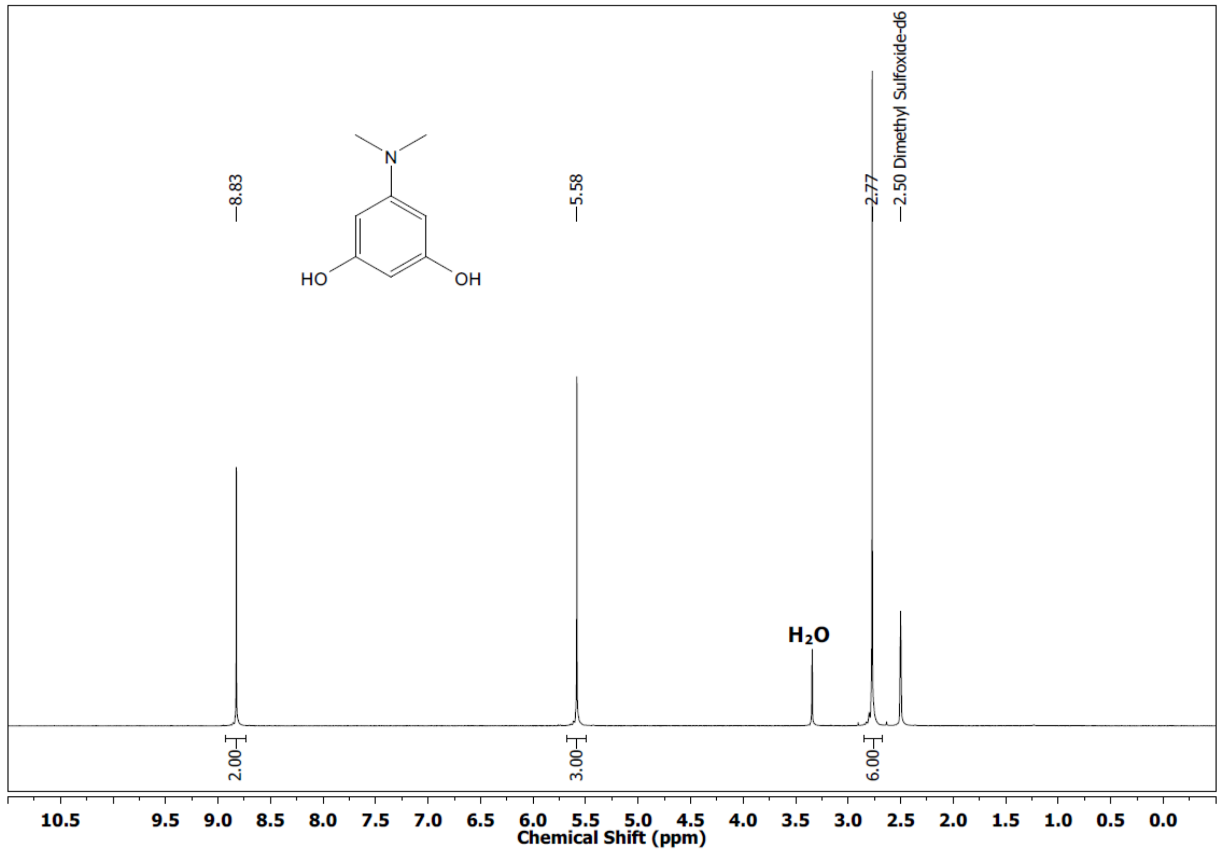

^{1}H NMR spectrum of Q2:

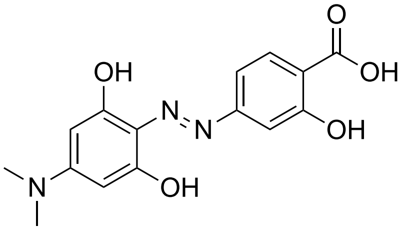

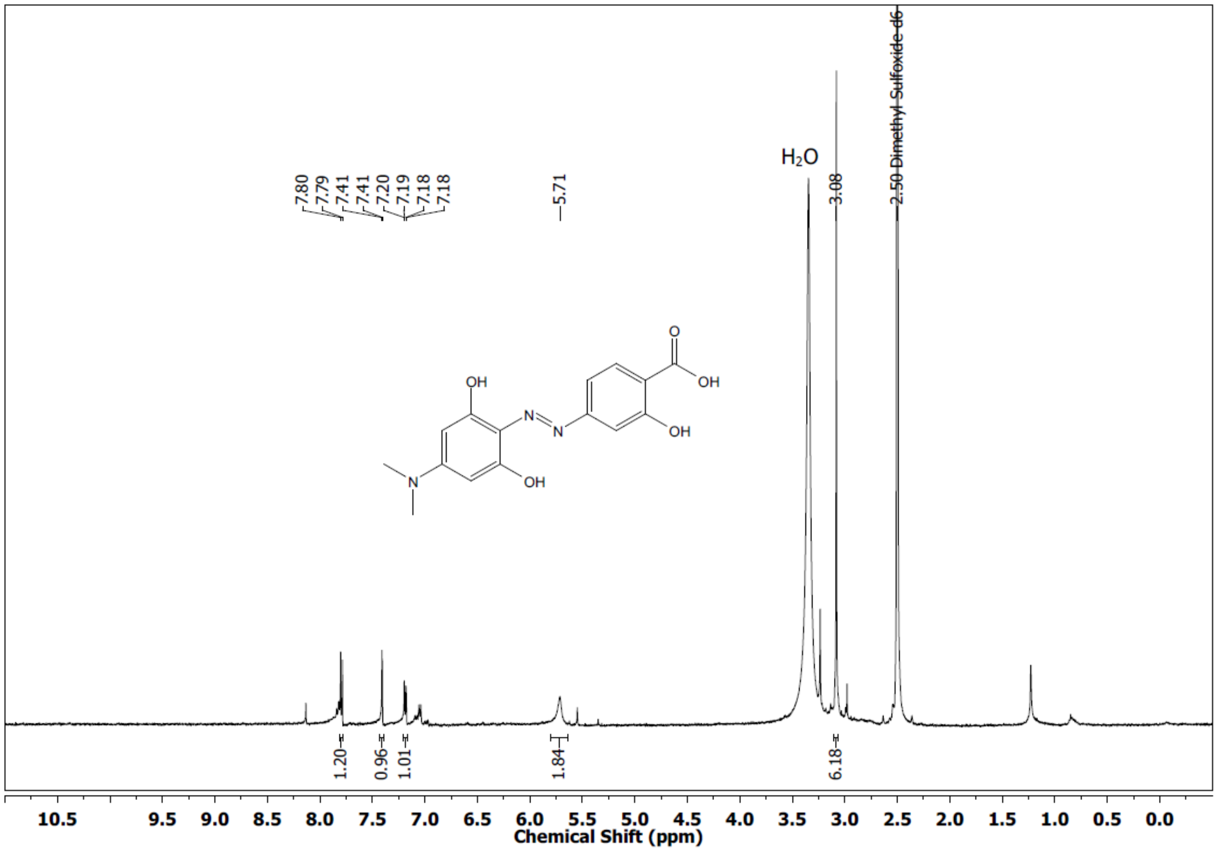

Supplementary References

1. Chen, Y.H., Chien, W.C., Lee, D.C. and Tan, K.T. Signal Amplification and Detection of Small Molecules via the Activation of Streptavidin and Biotin Recognition. *Analytical Chemistry*, **2019**, 91, 12461-12467.
2. Weinrich, T., Gränz, M., Grünewald, C., Prisner, T.F. and Göbel, M.W. Synthesis of a cytidine phosphoramidite with protected nitroxide spin label for EPR experiments with RNA. *European Journal of Organic Chemistry*, **2017**, 3, 491-496.
3. Terai, T., Maki, E., Sugiyama, S., Takahashi, Y., Matsumura, H., Mori, Y. and Nagano, T. Rational development of caged-biotin protein-labeling agents and some applications in live cells. *Chemistry & Biology*, **2011**, 18, 1261-1272.
4. Cai, B., Kim, D., Akhand, S., Sun, Y., Cassell, R.J., Alpsoy, A., Dykhuizen, E.C., Van Rijn, R.M., Wendt, M.K. and Krusemark, C.J. Selection of DNA-encoded libraries to protein targets within and on living cells. *Journal of the American Chemical Society*, **2019**, 141, 17057-17061.
5. Kempf, O., Kempf, K., Schobert, R. and Bombarda, E. Hydrodabcyl: a superior hydrophilic alternative to the dark fluorescence quencher dabcyl. *Analytical Chemistry*, **2017**, 89, 11893-11897.
